# Syndromic Craniosynostosis Can Define New Candidate Genes for Suture Development or Result from the Non-specifc Effects of Pleiotropic Genes: Rasopathies and Chromatinopathies as Examples

**DOI:** 10.3389/fnins.2017.00587

**Published:** 2017-10-18

**Authors:** Marcella Zollino, Serena Lattante, Daniela Orteschi, Silvia Frangella, Paolo N. Doronzio, Ilaria Contaldo, Eugenio Mercuri, Giuseppe Marangi

**Affiliations:** ^1^Institute of Genomic Medicine, Catholic University, A. Gemelli Hospital, Rome, Italy; ^2^Institute of Pediatric Neurology, Catholic University, A. Gemelli Hospital, Rome, Italy

**Keywords:** craniosynostosis, chromatinopathies, neurocristopathies, RASopathies, Kabuki syndrome, Koolen-De-Vries syndrome, Mowat–Wilson syndrome, Bohring-Opitz syndrome

## Abstract

Craniosynostosis is a heterogeneous condition caused by the premature fusion of cranial sutures, occurring mostly as an isolated anomaly. Pathogenesis of non-syndromic forms of craniosynostosis is largely unknown. In about 15–30% of cases craniosynostosis occurs in association with other physical anomalies and it is referred to as syndromic craniosynostosis. Syndromic forms of craniosynostosis arise from mutations in genes belonging to the Fibroblast Growth Factor Receptor (FGFR) family and the interconnected molecular pathways in most cases. However it can occur in association with other gene variants and with a variety of chromosome abnormalities as well, usually in association with intellectual disability (ID) and additional physical anomalies. Evaluating the molecular properties of the genes undergoing intragenic mutations or copy number variations (CNVs) along with prevalence of craniosynostosis in different conditions and animal models if available, we made an attempt to define two distinct groups of unusual syndromic craniosynostosis, which can reflect direct effects of emerging new candidate genes with roles in suture homeostasis or a non-specific phenotypic manifestation of pleiotropic genes, respectively. RASopathies and 9p23p22.3 deletions are reviewed as examples of conditions in the first group. In particular, we found that craniosynostosis is a relatively common component manifestation of cardio-facio-cutaneous (CFC) syndrome. Chromatinopathies and neurocristopathies are presented as examples of conditions in the second group. We observed that craniosynostosis is uncommon on average in these conditions. It was randomly associated with Kabuki, Koolen-de Vries/*KANSL1* haploinsufficiency and Mowat–Wilson syndromes and in *KAT6B*-related disorders. As an exception, trigonocephaly in Bohring-Opitz syndrome reflects specific molecular properties of the chromatin modifier *ASXL1* gene. Surveillance for craniosynostosis in syndromic forms of intellectual disability, as well as ascertainment of genomic CNVs by array-CGH in apparently non-syndromic craniosynostosis is recommended, to allow for improvement of both the clinical outcome of patients and the accurate individual diagnosis.

## Introduction

The premature fusion of cranial sutures affects ~1 in 2,500 newborns in a condition known as craniosynostosis (Cohen and MacLean, 2000; Boulet et al., 2008). Craniosynostosis appears to be a highly heterogeneous condition, that can be caused by different genetic defects or by environmental injuries (Lattanzi et al., 2012, 2017). It occurs as an isolated anomaly in about 70–85% of cases (Wilkie et al., 2010; Greenwood et al., 2014; Heuzé et al., 2014), which are referred to as non-syndromic craniosynostosis. Both environmental factors, such as intrauterine fetal head constraint and prenatal valproate exposure, and gene variants following an oligogenic or monogenic pattern of pathogenesis, were shown to predispose to non-syndromic craniosynostosis (Hunenko et al., 2001; Heuzé et al., 2014). Of relevance, several of these gene variants involve transcription factors, growth factor receptors, including the FGFR family, and cytokines, that play an important role in bone morphogenesis.

In about 15–30% of cases craniosynostosis occurs in association with other physical anomalies, as a consequence of variable gene variants and different chromosome abnormalities and they are referred to as syndromic craniosynostosis. More than 180 craniosynostosis syndrome are currently known (Winter and Baraitser, 2011; McKusick and Hamosh, 2014). The most commonly mutated genes in syndromic craniosynostosis include *FGFR2, FGFR3*, and *FGFR1*, comprising the FGFR family (Johnson and Wilkie, 2011). The FGFRs bind to Fibroblast Growth Factor (FGF), promoting growth and differentiation of mesenchymal and neuroectodermal cells. At a macroscopic level, FGFRs control cranial suture fusion. Using animal models, it has been demonstrated that mutated FGFRs lead to a defective FGF signal transduction which causes growth arrest of the cranium and the midface (Passos-Bueno et al., 2008; Holmes and Basilico, 2012).

Other genes with roles in the same morphogenic events regulated by the FGFR family have been described in craniosynostosis, including *TWIST1* (TWIST family bHLH transcription factor 1; ^*^601622), *EFNB1*(Ephrin B1; ^*^300035)*, POR* (Cytochrome P450 oxidoreductase; ^*^124015)*, RAB23* (RAS-associated protein RAB23; ^*^606144), and *EFNA4* (Ephrin A4; ^*^601380) (Merrill et al., 2006; Wilkie et al., 2007; Melville et al., 2010; Jezela-Stanek and Krajewska-Walasek, 2013).

On the other hand a variety of gene mutations outside the FGFR family (Twigg and Wilkie, 2015) and different chromosome abnormalities as well (including submicroscopic aberrations), such as trisomy 21, del (17q21.31) and dup(22q11) (Wilkie et al., 2010), have been consistently associated with syndromic craniosynostosis. A question of debate is whether craniosynostosis in these heterogeneous conditions can suggest new candidate genes, or it simply represents a non-specific effect of pleiotropic genes. Of relevance for possible targeted therapy, both non-syndromic and syndromic forms of craniosynostosis most likely share common molecular pathways.

In the present paper we have attempted to define two distinct groups of unusual syndromic craniosynostosis not related to mutations in genes coding for FGFRs, according to this different hypothesis of pathogenesis. Criteria for definition were molecular properties of the genes undergoing intragenic mutations or CNVs; prevalence of craniosynostosis in the reported conditions; animal models if available. The present review is not exhaustive for all syndromic forms of craniosynostosis.

## Gene-related syndromic craniosynostosis

RASopathies and 9p23p22.3 deletions are reviewed as examples of conditions in this group.

### RASopathies

RASopathies or RAS/mitogen-activated protein kinase (MAPK) syndromes are conditions caused by germline mutations in several genes encoding proteins of the RAS/MAPK signaling pathway characterized by overlapping phenotypes (Aoki et al., 2016). These disorders include neurofibromatosis type I, Legius syndrome, Noonan syndrome, Noonan syndrome with multiple lentigines (formerly called LEOPARD syndrome), Costello syndrome, cardiofaciocutaneous (CFC) syndrome, Noonan-like syndrome, hereditary gingival fibromatosis and capillary malformation–arteriovenous malformation. Beside many overlapping features, unique characteristics and specific genes are usually associated with each disorder.

Noonan syndrome (NS, OMIM 163950) is characterized by short stature, distinctive craniofacial features including a webbed or short neck, hypertelorism, downslanting palpebral fissures, ptosis, and low-set ears, congenital heart defects including pulmonary valve stenosis and hypertrophic cardiomyopathy, bleeding and myeloproliferative disorders and mild neurocognitive delay (Aoki and Matsubara, 2013). NS is caused by mutations in *PTPN11* (Protein Tyrosine Phosphatase, Non-Receptor Type 11, ^*^176876), *SOS1* (SOS Ras/Rac Guanine Nucleotide Exchange Factor 1, ^*^182530), *RAF1* (Raf-1 Proto-Oncogene, Serine/Threonine Kinase, ^*^164760), *KRAS* (KRAS Proto-Oncogene, GTPase, ^*^190070), *BRAF* (B-Raf Proto-Oncogene, Serine/Threonine Kinase, ^*^164757), *SHOC2* (SHOC2, Leucine Rich Repeat Scaffold Protein, ^*^602775), *RIT1* (Ras Like Without CAAX 1, ^*^609591), *NRAS* (NRAS Proto-Oncogene, GTPase, ^*^164790), *RRAS* (Related RAS Viral (R-Ras) Oncogene Homolog, ^*^165090), *RASA2* (RAS P21 Protein Activator 2, ^*^601589), *A2ML1* (Alpha-2-Macroglobulin Like 1, ^*^610627), *SOS2* (SOS Ras/Rho Guanine Nucleotide Exchange Factor 2, ^*^611247), and *LZTR1* (Leucine Zipper Like Transcription Regulator 1, ^*^600574), although it is estimated that about 20% of the causative genes behind NS are still unidentified. Cardiofaciocutaneous (CFC, OMIM 115150) is characterized by failure to thrive, distinctive facial features with high forehead and bitemporal constriction, ectodermal abnormalities, including palmoplantar keratosis, congenital heart diseases and severe psychomotor retardation. CFC syndrome is caused by mutations in *BRAF* (B-Raf Proto-Oncogene, Serine/Threonine Kinase ^*^164757) (about 70% of cases), *MAP2K1* (Mitogen-activated protein kinase kinase, ^*^176872) and *KRAS* (KRAS Proto-Oncogene, GTPase, ^*^190070).

Among RASopathies, craniosynostosis appears to be consistently associated with NS and CFC syndrome only, and with mutations limited to *PTPN11, SHOC2, KRAS*, and *BRAF* (Takenouchi et al., 2014; Addissie et al., 2015; Ueda et al., 2017). In the Ueda et al. report (2017), 3 out of 34 NS patients (9%) and 6 out of 18 CFC patients (33%) had craniosynostosis, and craniosynostosis affected all patients with mutations in *KRAS*. This strong genotype-phenotype association suggests specific mechanisms of pathology, worthy of investigation.

There is an interaction between FGFR and RAS/MAPK signaling pathways, as demonstrated by experiments in an FGFR mouse model where craniosynostosis is rescued using an inhibitor of RAS/MAPK signaling and by the fact that mutations in *ERF* (ETS2 repressor factor, ^*^611888, a gene at the end of the FGFR-RAS/MAPK cascade) also cause craniosynostosis (Shukla et al., 2007; Takenouchi et al., 2014; Addissie et al., 2015). FGFRs act upstream of the RAS/MAPK signaling pathway, and some proteins participating in the RAS/MAPK signaling were observed to partially mediate dysregulated cranial development caused by mutations in *FGFR* genes (Shukla et al., 2007).

### 9p23p22.3 deletion and trigonocephaly

The incidence of trigonocephaly is 1:15,000 live births (Kimonis et al., 2007). The etiology is still unknown but it is well established that an association with chromosomal abnormalities exists, especially with monosomy 9p syndrome (OMIM 158170). Deletion of the 9p23p22.3 region gives rise to a contiguous gene syndrome characterized by intellectual disability, distinctive craniofacial dysmorphism including upslanting palpebral fissures, hypertelorism, epicanthal folds, small palpebral fissures, flat nasal bridge, long philtrum, micrognatia and midface hypoplasia, congenital heart defect, and trigonocephaly. Trigonocephaly/prominent forehead is described in about 80% of cases (Huret et al., 1988; Swinkels et al., 2008).

An analysis of the gene content of CNVs affecting the 9p22.3 region, as reported by Vissers et al. (2011), indicated that *FREM1* (FRAS1-related extracellular matrix protein 1; ^*^608944) could potentially be a major gene associated with trigonocephaly, through haploinsufficiency. Of note, micro-computed tomography based analyses of the human equivalent mouse suture revealed advanced fusion in mice homozygous for Frem1 mutant alleles (Vissers et al., 2011). However further studies are required to verify the association of *FREM1* mutation and craniosynostosis.

Furthermore, it was suggested that trigonocephaly associated with 9p23p22.3 deletions is most likely oligogenic in pathogenesis. The receptor-type protein tyrosine phosphatase gene (*PTPRD*) was defined as another candidate gene for trigonocephaly by overlapping analysis of different chromosome deletions (Mitsui et al., 2013). PTPRD belongs to the protein tyrosine phosphatase family, playing essential roles in the regulation of receptor tyrosine kinase, growth, cell migration, and angiogenesis (Ortiz et al., 2014).

## Syndromic craniosynostosis as a nonspecific feature of conditions caused by mutations in pleiotropic genes

Chromatinopathies and neurocristopathies are discussed in detail as examples of conditions in this group. Many chromosomal disorders with low frequency occurrence of craniosynostosis can be included in this group as well.

### Chromatinopathies

Chromatinopathies refer to a highly heterogeneous group of syndromic conditions also defined as mendelian disorders of chromatin modification, in which the underlying genetic anomaly consists of disruption of one of the components of the epigenetic machinery. Targets of epigenetic modifications can be the DNA itself, through cytosine methylation; the methylation status is read by proteins that contain methyl-binding domains; DNA methylation of cytosines can be removed. Components of the epigenetic machinery targeting DNA include various enzymes with roles in each of these steps, thus acting as writers, readers, and erasers, respectively. Epigenetic modifications can target the DNA-associated histone proteins as well. Similar to DNA modifications, the histone machinery in this case consists of writers, readers, and erasers but also of remodelers, which have a role in balancing the open or compact status of the chromatin leading to transcription regulation (Fahrner and Bjornsson, 2014).

Chromatinopathies can be caused by mutations in genes in each category of the epigenetic machinery, including writers (i.e., Kabuki 1, Sotos, Kleefstra, Koolen-De-Vries*/KANSL1* haploinsufficiency, Rubinstein-Taybi, *KAT6B*-related syndromes); readers (i.e., Smith-Magenis, Rett syndromes), erasers (i.e., Kabuki 2, Townes-Brock, Bohring-Opitz syndromes) and chromatin remodellors (i.e., ATRX, CHARGE, Floating-Harbor syndromes). About 44 Mendelian disorders of the epigenetic machinery have been described so far (reviewed by Bjornsson, 2015).

Chromatinopathies are characterized by distinctive features, both genetically and clinically. First, mutations affecting the epigenetic machinery are expected to have widespread downstream epigenetic consequences, accounting for great pleiotropy of the genetic defect. Supporting this concept, although the most frequent clinical manifestation is intellectual disability, suggesting that maintenance of the normal epigenotype is important for neuronal homeostasis, a wide variety of additional anomalies can occur, including limb malformations, disorders of the neuronal migration, immune dysfunction, growth impairment and skeletal anomalies. Craniosynostosis has been described as a consistent, although unusual, feature of 4 out of 44 Mendelian disorders of the epigenetic machinery (reviewed by Bjornsson, 2015). One can speculate that the final phenotype in individual patients can reflect not only balance disruption of the different compartments of the epigenetic machinery (Paro, 1995), but also the molecular constitution of the target genes (Law et al., 2010).

All these reasons can likely account for the usually low frequency of craniosynostosis in chromatinopathies and for its association with specific conditions as well.

To the best of our knowledge, craniosynostosis has been described in some patients with Kabuki syndrome, Koolen-De-Vries/*KANSL1* haploinsufficiency syndrome, Bohring-Opitz syndrome and *KAT6B*-related disorders.

### Kabuki syndrome (KS)

Kabuki syndrome (KS, OMIM 147920) is a rare monogenic condition characterized clinically by distinctive facial dysmorphisms featuring the makeup used in traditional Japanese Kabuki theater, with arched eyebrows, long eyelashes and everted lower lids (Niikawa et al., 1988). Additional features include intellectual disability of different degrees, short stature, persistent fingertip pads and skeletal abnormalities. Congenital heart defects (CHDs) represent another important component manifestation. KS can be caused by mutations in *KMT2D* (Lysine-specific methyltransferase 2D, previously *MLL2*, ^*^602113), most frequently (referred to as KS1) (Ng et al., 2010), or in *KDM6A* (Lysine-specific demethylase 6A, ^*^300128) (referred to as KS2) (Lederer et al., 2012; Miyake et al., 2013). KMT2D is a component of the SET-domain-containing family of histone methyltransferases, enzymes that trimethylates histone H3 at lysine 4 (H3K4me3), in transcriptionally active genes (Smith et al., 2011). On the contrary, KDM6A is involved in gene silencing by removal of H3K27me3 mark (Hübner and Spector, 2011). Thus, both proteins act in promoting transcription of downstream genes through epigenetic modifications.

Several literature reports support the evidence that craniosynostosis is a component manifestation of the KS phenotype, emphasizing the importance of the accurate clinical assessment of children with craniosynostosis. In the literature, the prevalence of craniosynostosis in KS is about 6% (Armstrong et al., 2005; Topa et al., 2017). *KMT2D* was tested in one patient only, with positive results (Topa et al., 2017).

### Koolen-de vries/KANSL1 haploinsufficiency syndrome

*KANSL1* (KAT8 regulatory NSL complex, subunit 1) haploinsufficiency syndrome, also referred to as Koolen–De Vries syndrome (OMIM 610443), is characterized by highly typical facial features, including long and prominent philtrum, pear-shaped nose, everted lower lip and sparse eyebrows, mild-to-moderate intellectual disability, hypotonia and friendly behavior. Epilepsy, kidney anomalies and heart defects are also detected in about half of the patients. This condition is quite prevalent among ID patients, and it affects about 1:16,000 subjects among general population (Koolen et al., 2008). It was first described as a genomic disorder caused by a recurrent 0.450–0.600 Mb deletion on chromosome 17q21.31. However loss-of-function mutations in the *KANSL1* gene, residing in the deletion interval, were demonstrated to be sufficient in causing a full clinical phenotype (Koolen et al., 2012; Zollino et al., 2012).

KANSL1 is a member of a histone acetyltransferase complex that plays important roles in transcription regulation of many genes by acetylating histone H4 on lysine 16 (H4K16). Acetylation of H4K16 is known to play a pivotal role in transcription stimulation by inhibiting the compaction of 30 nm chromatin fibers. KANSL1 is also capable of acetylating non-histone substrates, namely p53 protein on lysine 120, and KANSL1 is essential for the transcription of p53 target genes (Huang et al., 2012).

We recently characterized both clinically and genetically a cohort of 32 patients with *KANSL1* haploinsufficiency syndrome, of whom 27 had a 17q21.31 deletion, and 5 an intragenic *KANSL1* mutation (Zollino et al., 2015). One of these cases had scaphocephaly, which required surgical correction at age 3 months.

We further analyzed 10 novel patients with *KANSL1* haploinsufficiency syndrome, of whom 9 had a 17q21.31 deletion and one a *de novo* intragenic mutation in *KANSL1* (c.1652+2 T>C; p.L552FfsX14). Notably, one patient with a chromosome deletion had sagittal craniosynostosis, which was surgically corrected at 4 months. Detailed information are part of a forthcoming clinically oriented paper.

Overall, craniosynostosis affected 2 out of 42 patients in our cohort (5%), who both underwent direct sequencing of *FGFR2, FGFR3, FGFR1* and *TWIST1*, with normal results. It occurred in association with 17q21.31 deletion in both patients.

Craniosynostosis has been described also in other patients carrying a 17q21.31 microdeletion. Specifically, clinical signs reported in these patients consist of sagittal synostosis (Koolen et al., 2008; Sharkey et al., 2009), scaphocephaly (Dubourg et al., 2011; Koolen et al., 2016) and in general an abnormally shaped skull (dolichocephaly, metopic ridge, bitemporal narrowing, trigonocephaly, brachycephaly, frontal bossing) (Koolen et al., 2016). It was demonstrated that loss-of-function mutations in *KANSL1* are sufficient to cause the full clinical phenotype associated with 17q21.31 deletions. Accordingly, whether craniosynostosis can reflect haploinsufficiency of contiguous genes in this region is questionable.

### KAT6B-related disorders

The gene *KAT6B* (Lysine acetyltransferase 6B, ^*^605880) encodes a histone acetyltransferase. This protein, which is part of the MOZ/MORF complex, also plays a role in transcriptional activation and repression and could be involved in brain development. *KAT6B-*related disorders include a great spectrum of conditions caused by heterozygous mutations in *KAT6B* (Clayton-Smith et al., 2011). These conditions were first described as separate entities, including Say-Barber-Biesecker-Young-Simpson syndrome (SBBYSS, OMIM 603736) and its variant Ohdo syndrome (OMIM 249620), and genitopatellar syndrome (GTPTS, OMIM 606170). *KAT6B-*related disorders are characterized by intellectual disability and by a variable association of additional features, including a distinctive facial dysmorphism with mask-like face, blepharophimosis, ptosis, broad nasal tip, dislocated or hypoplastic patellae and skeletal anomalies. Recently, 2 patients with sagittal craniosynostosis and clinical manifestations consistent with the Lin-Gettig syndrome phenotype have been described as carriers of *de novo* frameshift mutations in *KAT6B*. Interestingly, both patients had features overlapping those of SBBYSS and GTPTS (Bashir et al., 2017), suggesting that Lin-Gettig syndrome is in the spectrum of *KAT6B*-related disorders as well.

### Bohring-opitz syndrome

As exception among chromatinopathies, in Bohring-Opitz syndrome (BOPS; OMIM 605039) craniosynostosis rather reflects specific molecular properties of the causative gene.

BOPS is a malformation syndrome characterized by severe intrauterine growth retardation, intellectual disability, and a constellation of highly distinctive additional anomalies, including exophthalmos, nevus flammeus of the face and joints deviation of upper limbs. A key diagnostic feature in BOPS is trigonocephaly, described in 90% of patients (Hoischen et al., 2011; Magini et al., 2012; Dangiolo et al., 2015). In 2011, *ASXL1* was identified as the causative gene of this condition (Hoischen et al., 2011). *De novo* heterozygous mutations, which are mostly non-sense or truncating in nature, are detected in most patients. The gene *ASXL1* (Additional sex combs-like 1, ^*^612990) is involved in the activation and silencing of the *HOX* genes and in chromatin remodeling (Hoischen et al., 2011). Trigonocephaly was detected in 9/10 BOPS patients with a proven mutation in *ASXL1* (Hoischen et al., 2011; Magini et al., 2012; Dangiolo et al., 2015) and in 28/30 patients not tested for this gene (Hastings et al., 2011).

## Neurocristopathies

### Mowat–wilson syndrome

Mowat–Wilson syndrome (MWS; OMIM 235730) is a haploinsufficiency syndrome caused by heterozygous deletions or mutations in the *ZEB2* gene. In addition to moderate to severe ID, typical clinical features are craniofacial anomalies with hypertelorism, deep-set eyes, broad and medially flared eyebrows, wide nasal bridge, prominent nasal tip and columella, M-shaped upper lip, pointed triangular chin, linearized mandibular bones, cupped ears with upturned lobules and microcephaly. In addition, MWS patients have moderate-to-severe intellectual disability, epilepsy, Hirschsprung disease and multiple congenital anomalies, covering genital anomalies, congenital heart disease and agenesis of the corpus callosum. Over 200 patients have been described so far with a proven loss-of-function variant in *ZEB2* (Dastot-Le Moal et al., 2007; Garavelli and Mainardi, 2007; Garavelli et al., 2009; Zollino et al., 2011). The heterogeneous basic genomic defect consists in chromosome 2q21-q23 deletions in a few patients, and in variable loss-of-function intragenic mutations in most. The *ZEB2* gene (Zinc finger E box-binding homeobox 2, ^*^605802) is a member of the ZFH1 (ZEB) family of two-handed zinc finger/homeodomain proteins, which are transcription factors playing an important role during embryonic development (Verschueren et al., 1999). In particular, they are involved in the “epithelial to mesenchymal transition” (EMT) process, permitting epithelial cells to undergo mesenchymal differentiation, which is essential for many morphogenetic events. The role of *ZEB2* in cranial suture development is not clear and needs to be addressed at a molecular level. However, it may be related to any of the following aspects: (a) ZEB2 interaction with Smad transcription factors implies an involvement in the TGFβ signaling pathway, which plays an important role in modulating suture fusion and includes some proteins already known to be disrupted in some forms of craniosynostosis; (b) ZEB2 acts to repress mesoendodermal genes to induce neuroectoderm formation, and dysregulation of this process might affect the suture mesoderm and result in bad fusion timing; (c) ZEB2 is also a regulator of neural crest cell epithelial-mesenchymal transition, a role that it shares with TWIST, an important regulator of cranial suture development during embryogenesis and haploinsufficiency of which causes Saethre-Chotzen syndrome (Hegarty et al., 2015). The use of animal models, such as Xenopus (van Grunsven et al., 2007), mice (Van de Putte et al., 2003), and studies on human cells as well (Espinosa-Parrilla et al., 2002), demonstrated that ZEB2 is implicated in neuroectoderm development. In human embryos, ZEB2 is widely expressed in all the central nervous system, in facial neuroctoderm, in craniofacial bones and in the middle ear region. Defective expression in these areas corresponds to specific clinical signs, such as agenesis of corpus callosum (diencephalon), epilepsy (mesencephalon), intellectual disability (rhombencephalon), facial dysmorphisms (facial neuroctoderm), abnormal mandibular bones (craniofacial bones), typical ear conformation (ear region surrounding the developing ossicles).

It is worth stating that the ablation of the neural crest precursor-specific gene Zfhx1b (Zinc finger homeobox 1B, alternative name for *ZEB2*) in mice leads to many malformations reflecting the MWS phenotype (Van de Putte et al., 2003). Based on this evidence, MWS can be considered a syndromic form of neurocristopathy.

In the literature, craniosynostosis has been described in 4 out of 214 MWS cases (1.9%; Adam et al., 2008; Hartill et al., 2014; Wenger et al., 2015). Thus, craniosynostosis appears to be unusual in MWS patients, raising the question as to whether it represents only a coincidental finding. However, craniosynostosis in MWS is linked to *ZEB2* haploinsufficiency, most likely, as we previously suggested (Wenger et al., 2015).

Syndromic conditions with gene-related craniosynostosis, or with craniosynostosis as a non-specific effect of pleiotropic genes, are summarized in Table 1.

**Table 1:** Examples of unusual syndromic forms of craniosynostosis reflecting the pathogenic role of new candidate genes (Group 1) or nonspecific effects of pleiotropic genes (Group 2).

		**Patients**		**References**
		***n***	**%**	
**GROUP 1**				
**Gene-related syndromic craniosynostosis**				
RASopathies				
*PTPN11*	Noonan syndrome	3/34	9%	Ueda et al., 2017
*KRAS*	Cardiofaciocutaneous syndrome or Noonan syndrome	12/80	15%	Addissie et al., 2015; Ueda et al., 2017
*BRAF*	Cardiofaciocutaneous syndrome	4/10	40%	Ueda et al., 2017
*9p23p22.3 deletions*				
*FREM1*		3/104^*^	2.9%	Vissers et al., 2011
*PTPRD*		1		Choucair et al., 2015
**GROUP 2**				
**Craniosynostosis as non-specific sign of pleiotropic genes**				
Chromatinopathies				
*del 17q21.31 (KANSL1 haploinsufficiency)*	Koolen-de Vries syndrome	14/116	12%	Koolen et al., 2008; Sharkey et al., 2009; Dubourg et al., 2011 Zollino et al., 2015 and personal data; Koolen et al., 2016
KMT2D^**^	Kabuki syndrome	3/486 ^***^	6%	Gillis et al., 1990; Ewart-Toland et al., 1998 David et al., 2004; Geneviève et al., 2004 Armstrong et al., 2005 Martínez-Lage et al., 2010 Topa et al., 2017
*KAT6B*	KAT6B-related disorders	2/36	5.5%	Gannon et al., 2015; Bashir et al., 2017
*ASXL1*	Bohring-Opitz syndrome	9/10^****^	90%	Hoischen et al., 2011; Magini et al., 2012; Dangiolo et al., 2015
Neurocristopathies				
*ZEB2*	Mowat–Wilson syndrome	4/214	1.9%	Adam et al., 2008; Hartill et al., 2014; Wenger et al., 2015

* *These variants have been identified also in control individuals and they have been reported in different databases (ExAC; gnomAD) so their pathogenic relevance remains uncertain*.

** *KMT2D tested in only one out of 9 Kabuki syndrome patients with craniosynostosis, with positive results (Topa et al., 2017)*.

*** *Single case reports. In only one paper (Geneviève et al., 2004), craniosynostosis is described to affect 1/20 KS patients (5% of cases)*.

**** *Trigonocephaly has been described also in an additional 28/30 Bohring-Opitz syndrome patients who received a clinically based diagnosis of this condition (Hastings et al., 2011). As an exception, this disorder of the epigenetic machinery reflects specific gene properties (see text)*.

## Concluding remarks

Syndromic forms of craniosynostosis are clinically and genetically heterogeneous, with genomic defects including both quantitative chromosome abnormalities and intragenic mutations. Most cases arise from disruption of the morphogenic events regulated by the FGFR family proteins and their interconnected signaling pathways.

However, mutations in other genes not involved in these pathways and several chromosome abnormalities have been consistently associated with craniosynostosis, reflecting either a gene-related mechanism or pleiotropy of the mutated genes. Among the first group, RASopathies, Bohring-Opitz syndrome, and selected chromosome alterations, such as 9p23p22.3 deletions, can allow for definition of new candidate genes and for likely targeted therapy. On the other hand, craniosynostosis in chromatinopathies and neurocristopathies, and in many chromosome alterations as well, rather reflects pleiotropy of the associated genes, leading to widespread impairment of gene transcription.

Although, most non-FGFR-related syndromic conditions with craniosynostosis are associated with intellectual disability and physical dysmorphisms, both cognitive impairment and dysmorphisms can be very mild. Furthermore, cognitive impairment, usually of very mild degree, can affect a subset of individuals with non-syndromic craniosynostosis. On this evidence, ascertainment of additional signs by extensive clinical evaluation of all patients with craniosynostosis is recommended, and a wider application of whole genome investigations, namely array-CGH, is recommended as well.

Finally, surveillance for craniosynostosis could be planned in many syndromic forms of intellectual disability, for the benefit of early diagnosis and early therapy.

## Author contributions

MZ conceived the studies and wrote the manuscript. SL and GM analyzed data and wrote the manuscript. DO, SF, and PD performed experiments. IC and EM recruited patients. All authors read and approved the final manuscript.

### Conflict of interest statement

The authors declare that the research was conducted in the absence of any commercial or financial relationships that could be construed as a potential conflict of interest.

## References

[B1] Adam M. P. Justice A. N. Bean L. J. Fernhoff P. M. (2008). Mowat-Wilson syndrome with craniosynostosis: a case report. Am. J. Med. Genet. A. 146A, 245–246. 10.1002/ajmg.a.32075 18076118

[B2] Addissie Y. A. Kotecha U. Hart R. A. Martinez A. F. Kruszka P. Muenke M. (2015). Craniosynostosis and Noonan syndrome with KRAS mutations: expanding the phenotype with a patient report and review of the literature. Am. J. Med. Genet. A. 167A, 2657–2663. 10.1002/ajmg.a.37259 26249544

[B3] Aoki Y. Matsubara Y. (2013). Ras/MAPK syndromes and childhood hemato-oncological diseases. Int. J. Hematol. 97, 30–36. 10.1007/s12185-012-1239-y 23250860

[B4] Aoki Y. Niihori T. Inoue S. Matsubara Y. (2016). Recent advances in RASopathies. J. Hum. Genet. 61, 33–39. 10.1038/jhg.2015.114 26446362

[B5] Armstrong L. Abd El Moneim A. Aleck K. Aughton D. J. Baumann C. Braddock S. R. (2005). Further delineation of Kabuki syndrome in 48 well-defined new individuals. Am. J. Med. Genet. A. 132, 265–272. 10.1002/ajmg.a.30340 15690370

[B6] Bashir R. A. Dixit A. Goedhart C. Parboosingh J. S. Innes A. M. Care for Rare Canada Consortium. (2017). Lin-Gettig syndrome: Craniosynostosis expands the spectrum of the KAT6B related disorders. Am. J. Med. Genet. A 173, 2596–2604. 10.1002/ajmg.a.38355 28696035

[B7] Bjornsson H. T. (2015). The Mendelian disorders of the epigenetic machinery. Genome Res. 25, 1473–1481. 10.1101/gr.190629.115 26430157 PMC4579332

[B8] Boulet S. L. Rasmussen S. A. Honein M. A. (2008). A population-based study of craniosynostosis in metropolitan Atlanta, 1989-2003. Am. J. Med. Genet. A 146A, 984–991. 10.1002/ajmg.a.32208 18344207

[B9] Choucair N. Mignon-Ravix C. Cacciagli P. Abou Ghoch J. Fawaz A. Mégarbané A.. (2015). Evidence that homozygous PTPRD gene microdeletion causes trigonocephaly, hearing loss, and intellectual disability. Mol. Cytogenet. 8:39. 10.1186/s13039-015-0149-0 26082802 PMC4469107

[B10] Clayton-Smith J. O'Sullivan J. Daly S. Bhaskar S. Day R. Anderson B. (2011). Whole-exome-sequencing identifies mutations in histone acetyltransferase gene KAT6B in individuals with the Say-Barber-Biesecker variant of Ohdo syndrome. Am. J. Hum. Genet. 89, 675–681. 10.1016/j.ajhg.2011.10.008 22077973 PMC3213399

[B11] Cohen M. M. Jr. MacLean R. E. (eds.). (2000). Craniosynostosis: Diagnosis, Evaluation and Management, 2nd Edn. New York, NY: Oxford University Press.

[B12] Dangiolo S. B. Wilson A. Jobanputra V. Anyane-Yeboa K. (2015). Bohring-Opitz syndrome (BOS) with a new ASXL1 pathogenic variant: review of the most prevalent molecular and phenotypic features of the syndrome. Am. J. Med. Genet. A 167A, 3161–3166. 10.1002/ajmg.a.37342 26364555

[B13] Dastot-Le Moal F. Wilson M. Mowat D. Collot N. Niel F. Goossens M. (2007). ZFHX1B mutations in patients with Mowat-Wilson syndrome. Hum. Mutat. 28, 313–321. 10.1002/humu.20452 17203459

[B14] David G. Sillence D. Hardwick R. Opitz J. M. (2004). A case of Kabuki (Niikawa-Kuroki) syndrome associated with manifestations resembling C-trigonocephaly syndrome. Am. J. Med. Genet. A. 130A, 389–392. 10.1002/ajmg.a.20599 15389716

[B15] Dubourg C. Sanlaville D. Doco-Fenzy M. Le Caignec C. Missirian C. Jaillard S.. (2011). Clinical and molecular characterization of 17q21.31 microdeletion syndrome in 14 French patients with mental retardation. Eur. J. Med. Genet. 54, 144–151. 10.1016/j.ejmg.2010.11.003 21094706

[B16] Espinosa-Parrilla Y. Amiel J. Augé J. Encha-Razavi F. Munnich A. Lyonnet S.. (2002). Expression of the SMADIP1 gene during early human development. Mech. Dev. 114, 187–191. 10.1016/S0925-4773(02)00062-X 12175509

[B17] Ewart-Toland A. Enns G. M. Cox V. A. Mohan G. C. Rosenthal P. Golabi M. (1998). Severe congenital anomalies requiring transplantation in children with Kabuki syndrome. Am. J. Med. Genet. 80, 362–367. 10.1002/(SICI)1096-8628(19981204)80:4<362::AID-AJMG11>3.0.CO;2-W 9856564

[B18] Fahrner J. A. Bjornsson H. T. (2014). Mendelian disorders of the epigenetic machinery: tipping the balance of chromatin states. Annu. Rev. Genomics Hum. Genet. 15, 269–293. 10.1146/annurev-genom-090613-094245 25184531 PMC4406255

[B19] Gannon T. Perveen R. Schlecht H. Ramsden S. Anderson B. Kerr B.. (2015). Further delineation of the KAT6B molecular and phenotypic spectrum. Eur. J. Hum. Genet. 23, 1165–1170. 10.1038/ejhg.2014.248 25424711 PMC4351891

[B20] Garavelli L. Mainardi P. C. (2007). Mowat-Wilson syndrome. Orphan. J. Rare Dis. 2:42. 10.1186/1750-1172-2-42 17958891 PMC2174447

[B21] Garavelli L. Zollino M. Mainardi P. C. Gurrieri F. Rivieri F. Soli F.. (2009). Mowat-Wilson syndrome: facial phenotype changing with age: study of 19 Italian patients and review of the literature. Am. J. Med. Genet. A 149A, 417–426. 10.1002/ajmg.a.32693 19215041

[B22] Geneviève D. Amiel J. Viot G. Le Merrer M. Sanlaville D. Urtizberea A.. (2004). Atypical findings in Kabuki syndrome: report of 8 patients in a series of 20 and review of the literature. Am. J. Med. Genet. A 129A, 64–68. 10.1002/ajmg.a.30144 15266618

[B23] Gillis R. Klar A. Gross-Kieselstein E. (1990). The Niikawa-Kuroki (Kabuki make-up) syndrome in a Moslem Arab child. Clin. Genet. 38, 378–381. 10.1111/j.1399-0004.1990.tb03599.x 2282718

[B24] Greenwood J. Flodman P. Osann K. Boyadjiev S. A. Kimonis V. (2014). Familial incidence and associated symptoms in a population of individuals with nonsyndromic craniosynostosis. Genet. Med. 16, 302–310. 10.1038/gim.2013.134 24071792 PMC4143991

[B25] Hartill V. L. Pendlebury M. Hobson E. (2014). Mowat-Wilson syndrome associated with craniosynostosis. Clin. Dysmorphol. 23, 16–19. 10.1097/MCD.0000000000000016 24300291

[B26] Hastings R. Cobben J. M. Gillessen-Kaesbach G. Goodship J. Hove H. Kjaergaard S.. (2011). Bohring-Opitz (Oberklaid-Danks) syndrome: clinical study, review of the literature, and discussion of possible pathogenesis. Eur. J. Hum. Genet. 19, 513–519. 10.1038/ejhg.2010.234 21368916 PMC3083618

[B27] Hegarty S. V. Sullivan A. M. O'Keeffe G. W. (2015). Zeb2: a multifunctional regulator of nervous system development. Prog. Neurobiol. 132, 81–95. 10.1016/j.pneurobio.2015.07.001 26193487

[B28] Heuzé Y. Holmes G. Peter I. Richtsmeier J. T. Jabs E. W. (2014). Closing the gap: genetic and genomic continuum from syndromic to nonsyndromic craniosynostoses. Curr. Genet. Med. Rep. 2, 135–145. 10.1007/s40142-014-0042-x 26146596 PMC4489147

[B29] Hoischen A. van Bon B. W. Rodríguez-Santiago B. Gilissen C. Vissers L. E. de Vries P.. (2011). *De novo* nonsense mutations in ASXL1 cause Bohring-Opitz syndrome. Nat. Genet. 43, 729–731. 10.1038/ng.868 21706002

[B30] Holmes G. Basilico C. (2012). Mesodermal expression of Fgfr2S252W is necessary and sufficient to induce craniosynostosis in a mouse model of Apert syndrome. Dev. Biol. 368, 283–293. 10.1016/j.ydbio.2012.05.026 22664175 PMC3671595

[B31] Huang J. Wan B. Wu L. Yang Y. Dou Y. Lei M. (2012). Structural insight into the regulation of MOF in the male-specific lethal complex and the non-specific lethal complex. Cell Res. 22, 1078–1081. 10.1038/cr.2012.72 22547026 PMC3367523

[B32] Hübner M. R. Spector D. L. (2011). Role of H3K27 demethylases Jmjd3 and UTX in transcriptional regulation. Cold Spring Harb. Symp. Quant.Biol. 75, 43–49. 10.1101/sqb.2010.75.020 21209387

[B33] Hunenko O. Karmacharya J. Ong G. Kirschner R. E. (2001). Toward an understanding of nonsyndromic craniosynostosis: altered patterns of TGF-β receptor and FGF receptor expression induced by intrauterine head constraint. Ann. Plast. Surg. 46, 546–553. 10.1097/00000637-200105000-00015 11352430

[B34] Huret J. L. Leonard C. Forestier B. Rethore M. O. Lejeune J. (1988). Eleven new cases of del(9p) and features from 80 cases. J. Med. Genet. 25, 741–749. 10.1136/jmg.25.11.741 3070043 PMC1051577

[B35] Jezela-Stanek A. Krajewska-Walasek M. (2013). Genetic causes of syndromic craniosynostoses. Eur. J. Paediatr. Neurol. 17, 221–224. 10.1016/j.ejpn.2012.09.009 23062756

[B36] Johnson D. Wilkie A. O. (2011). Craniosynostosis. Eur. J. Hum. Genet. 19, 369–376. 10.1038/ejhg.2010.235 21248745 PMC3060331

[B37] Kimonis V. Gold J. A. Hoffman T. L. Panchal J. Boyadjiev S. A. (2007). Genetics of craniosynostosis. Semin. Pediatr. Neurol. 14, 150–161. 10.1016/j.spen.2007.08.008 17980312

[B38] Koolen D. A. Kramer J. M. Neveling K. Nillesen W. M. Moore-Barton H. L. Elmslie F. V.. (2012). Mutations in the chromatin modifier gene KANSL1 cause the 17q21.31 microdeletion syndrome. Nat. Genet. 44, 639–641. 10.1038/ng.2262 22544363

[B39] Koolen D. A. Pfundt R. Linda K. Beunders G. Veenstra-Knol H. E. Conta J. H.. (2016). The Koolen-de Vries syndrome: a phenotypic comparison of patients with a 17q21.31 microdeletion versus a KANSL1 sequence variant. Eur. J. Hum. Genet. 24, 652–659. 10.1038/ejhg.2015.178 26306646 PMC4930086

[B40] Koolen D. A. Sharp A. J. Hurst J. A. Firth H. V. Knight S. J. Goldenberg A.. (2008). Clinical and molecular delineation of the 17q21.31 microdeletion syndrome. J. Med. Genet. 45, 710–720. 10.1136/jmg.2008.058701 18628315 PMC3071570

[B41] Lattanzi W. Barba M. Di Pietro L. Boyadjiev S. A. (2017). Genetic advances in craniosynostosis. Am. J. Med. Genet. A 173, 1406–1429. 10.1002/ajmg.a.38159 28160402 PMC5397362

[B42] Lattanzi W. Bukvic N. Barba M. Tamburrini G. Bernardini C. Michetti F.. (2012). Genetic basis of single-suture synostoses: genes, chromosomes and clinical implications. Childs Nerv. Syst. 28, 1301–1310. 10.1007/s00381-012-1781-1 22872241

[B43] Law M. J. Lower K. M. Voon H. P. Hughes J. R. Garrick D. Viprakasit V.. (2010). ATR-X syndrome protein targets tandem repeats and influences allele-specific expression in a size-dependent manner. Cell 143, 367–378. 10.1016/j.cell.2010.09.023 21029860

[B44] Lederer D. Grisart B. Digilio M. C. Benoit V. Crespin M. Ghariani S. C.. (2012). Deletion of KDM6A, a histone demethylase interacting with MLL2, in three patients with kabuki syndrome. Am. J. Hum. Genet. 90, 119–124. 10.1016/j.ajhg.2011.11.021 22197486 PMC3257878

[B45] Magini P. Della Monica M. Uzielli M. L. Mongelli P. Scarselli G. Gambineri E.. (2012). Two novel patients with Bohring-Opitz syndrome caused by *de novo* ASXL1 mutations. Am. J. Med. Genet. A 158A, 917–921. 10.1002/ajmg.a.35265 22419483

[B46] Martínez-Lage J. F. Felipe-Murcia M. Navarro E. G. Almagro M. J. López-Guerrero A. L. Pérez-Espejo M. A. (2010). Craniosynostosis in Kabuki syndrome. J. Neurosurg. Pediatr. 6, 198–201. 10.3171/2010.5.PEDS09286 20672944

[B47] McKusick V. A. Hamosh A. (2014). OMIM. Available online for: http://www.omim.org

[B48] Melville H. Wang Y. Taub P. J. Jabs E. W. (2010). Genetic basis of potential therapeutic strategies for craniosynostosis. Am. J. Med. Genet. A 152A, 3007–3015. 10.1002/ajmg.a.33703 21082653

[B49] Merrill A. E. Bochukova E. G. Brugger S. M. Ishii M. Pilz D. T. Wall S. A.. (2006). Cell mixing at a neural crest-mesoderm boundary and deficient ephrin-Eph signaling in the pathogenesis of craniosynostosis. Hum. Mol. Genet. 15, 1319–1328. 10.1093/hmg/ddl052 16540516

[B50] Mitsui N. Shimizu K. Nishimoto H. Mochizuki H. Iida M. Ohashi H. (2013). Patient with terminal 9 Mb deletion of chromosome 9p. Refining the critical region for 9p monosomy syndrome with trigonocephaly. Congenit. Anom. 53, 49–53. 10.1111/j.1741-4520.2012.00362.x 23480358

[B51] Miyake N. Mizuno S. Okamoto N. Ohashi H. Shiina M. Ogata K.. (2013). KDM6A point mutations cause kabuki syndrome. Hum. Mutat. 34, 108–110. 10.1002/humu.22229 23076834

[B52] Ng S. B. Bigham A. W. Buckingham K. J. Hannibal M. C. McMillin M. J. Gildersleeve H. I.. (2010). Exome sequencing identifies MLL2 mutations as a cause of Kabuki syndrome. Nat. Genet. 42, 790–793. 10.1038/ng.646 20711175 PMC2930028

[B53] Niikawa N. Kuroki Y. Kajii T. Matsuura N. Ishikiriyama S. Tonoki H.. (1988). Kabuki make-up (Niikawa-Kuroki) syndrome: a study of 62 patients. Am. J. Med. Genet. 31, 565–589. 10.1002/ajmg.1320310312 3067577

[B54] Ortiz B. Fabius A. W. Wu W. H. Pedraza A. Brennan C. W. Schultz N.. (2014). Loss of the tyrosine phosphatase PTPRD leads to aberrant STAT3 activation and promotes gliomagenesis. Proc. Natl. Acad. Sci. U.S.A. 111, 8149–8154. 10.1073/pnas.1401952111 24843164 PMC4050622

[B55] Paro R. (1995). Propagating memory of transcriptional states. Trends Genet. 11, 295–297. 10.1016/S0168-9525(00)89081-2 8585123

[B56] Passos-Bueno M. R. Serti Eacute A. E. Jehee F. S. Fanganiello R. Yeh E. (2008). Genetics of craniosynostosis: genes, syndromes, mutations and genotype-phenotype correlations. Front. Oral Biol. 12, 107–143. 10.1159/000115035 18391498

[B57] Sharkey F. H. Morrison N. Murray R. Iremonger J. Stephen J. Maher E.. (2009). 17q21.31 microdeletion syndrome: further expanding the clinical phenotype. Cytogenet. Genome Res. 127, 61–66. 10.1159/000279260 20110647

[B58] Shukla V. Coumoul X. Wang R. H. Kim H. S. Deng C. X. (2007). RNA interference and inhibition of MEK-ERK signaling prevent abnormal skeletal phenotypes in a mouse model of craniosynostosis. Nat. Genet. 39, 1145–1150. 10.1038/ng2096 17694057

[B59] Smith E. Lin C. Shilatifard A. (2011). The super elongation complex (SEC) and MLL in development and disease. Genes Dev. 25, 661–672. 10.1101/gad.2015411 21460034 PMC3070929

[B60] Swinkels M. E. Simons A. Smeets D. F. Vissers L. E. Veltman J. A. Pfundt R.. (2008). Clinical and cytogenetic characterization of 13 Dutch patients with deletion 9p syndrome: delineation of the critical region for a consensus phenotype. Am. J. Med. Genet. A 146A, 1430–1438. 10.1002/ajmg.a.32310 18452192

[B61] Takenouchi T. Sakamoto Y. Miwa T. Torii C. Kosaki R. Kishi K.. (2014). Severe craniosynostosis with Noonan syndrome phenotype associated with SHOC2 mutation: clinical evidence of crosslink between FGFR and RAS signaling pathways. Am. J. Med. Genet. A 164A, 2869–2872. 10.1002/ajmg.a.36705 25123707

[B62] Topa A. Samuelsson L. Lovmar L. Stenman G. Kölby L. (2017). On the significance of craniosynostosis in a case of Kabuki syndrome with a concomitant KMT2D mutation and 3.2 Mbp *de novo* 10q22.3q23.1 deletion. Am. J. Med. Genet. A 173, 2219–2225. 10.1002/ajmg.a.38296 28590022

[B63] Twigg S. R. Wilkie A. O. (2015). A genetic-pathophysiological framework for Craniosynostosis. Am. J. Hum. Genet. 97, 359–377. 10.1016/j.ajhg.2015.07.006 26340332 PMC4564941

[B64] Ueda K. Yaoita M. Niihori T. Aoki Y. (2017). Craniosynostosis in patients with RASopathies: accumulating clinical evidence for expanding the phenotype. Am. J. Med. Genet. 173, 2346–2352. 10.1002/ajmg.a.38337 28650561

[B65] Van de Putte T. Maruhashi M. Francis A. Nelles L. Kondoh H. Huylebroeck D.. (2003). Mice lacking ZFHX1B, the gene that codes for Smad-interacting protein-1, reveal a role for multiple neural crest cell defects in the etiology of Hirschsprung disease-mental retardation syndrome. Am. J. Hum. Genet. 72, 465–470. 10.1086/346092 12522767 PMC379238

[B66] van Grunsven L. A. Taelman V. Michiels C. Verstappen G. Souopgui J. Nichane M.. (2007). XSip1 neuralizing activity involves the co-repressor CtBP and occurs through BMP dependent and independent mechanisms. Dev. Biol. 306, 34–49. 10.1016/j.ydbio.2007.02.045 17442301

[B67] Verschueren K. Remacle J. E. Collart C. Kraft H. Baker B. S. Tylzanowski P.. (1999). SIP1, a novel zinc finger/homeodomain repressor, interacts with Smad proteins and binds to 5'-CACCT sequences in candidate target genes. J. Biol. Chem. 274, 20489–20498. 10.1074/jbc.274.29.20489 10400677

[B68] Vissers L. E. Cox T. C. Maga A. M. Short K. M. Wiradjaja F. Janssen I. M.. (2011). Heterozygous mutations of FREM1 are associated with an increased risk of isolated metopic craniosynostosis in humans and mice. PLoS Genet. 7:e1002278. 10.1371/journal.pgen.1002278 21931569 PMC3169541

[B69] Wenger T. L. Harr M. Ricciardi S. Bhoj E. Santani A. Adam M. P. (2015). CHARGE-like presentation, craniosynostosis and mild Mowat-Wilson Syndrome diagnosed by recognition of the distinctive facial gestalt in a cohort of 28 new cases. Am. J. Med. Genet. A 167, 1682–1683. 10.1002/ajmg.a.36860 26097173

[B70] Wilkie A. O. Bochukova E. G. Hansen R. M. Taylor I. B. Rannan-Eliya S. V. Byren J. C.. (2007). Clinical dividends from the molecular genetic diagnosis of craniosynostosis. Am. J. Med. Genet. A 143A, 1941–1949. 10.1002/ajmg.a.31905 17621648

[B71] Wilkie A. O. Byren J. C. Hurst J. A. Jayamohan J. Johnson D. Knight S. J.. (2010). Prevalence and complications of single-gene and chromosomal disorders in craniosynostosis. Pediatrics 126:e391–400. 10.1542/peds.2009-3491 20643727 PMC3535761

[B72] Winter R. Baraitser M. (2011). Winter-Baraitser Dysmorphology Database, Version 3.0. London: Oxford University Press.

[B73] Zollino M. Garavelli L. Rauch A. (2011). Clinical utility gene card for: Mowat-Wilson syndrome. Eur. J. Hum. Genet. 19. 10.1038/ejhg.2011.12 21343952 PMC3172917

[B74] Zollino M. Marangi G. Ponzi E. Orteschi D. Ricciardi S. Lattante S.. (2015). Intragenic KANSL1 mutations and chromosome 17q21.31 deletions: broadening the clinical spectrum and genotype-phenotype correlations in a large cohort of patients. J. Med. Genet. 52, 804–814. 10.1136/jmedgenet-2015-103184 26424144

[B75] Zollino M. Orteschi D. Murdolo M. Lattante S. Battaglia D. Stefanini C.. (2012). Mutations in KANSL1 cause the 17q21.31 microdeletion syndrome phenotype. Nat. Genet. 44, 636–638. 10.1038/ng.2257 22544367

